# Drivers and barriers to career entry and retention of nurses with initial higher education: A scoping review

**DOI:** 10.3205/zma001759

**Published:** 2025-06-16

**Authors:** Julia Müller, Florian Breitinger, Patricia Bräuer, Bettina Dauer, Max Zilezinski, Denny Paulicke, Patrick Jahn

**Affiliations:** 1 Martin-Luther-University Halle-Wittenberg, Medical Faculty, Department of Internal Medicine, Working Group on Health Service Research, Nursing in Hospital, Halle (Saale), Germany; 2 Research Institute for Vocational Education and Training, Nürnberg and Potsdam, Germany; 3 Federal Institute for Vocational Education and Training (VET), Bonn, Germany; 4 Charité-University Medicine Berlin, Institute for Clinical Nursing Science Charité Centre 1 for Human and Health Sciences – Campus Charitè Mitte, Berlin, Germany; 5 Akkon University of Human Sciences, Department of Medical Education, Berlin, Germany

**Keywords:** initial higher education, staff development, nursing, barriers, drivers, scoping review

## Abstract

**Background:**

While previous systematic reviews have examined specific aspects of the career trajectories of bachelor-qualified nurses, including adjustment strategies, workplace challenges, and support programmes, no comprehensive synthesis exists addressing how these factors interact across the broader early career pathway.

This article examines the drivers and barriers influencing entry, role transition, and retention of graduate nurses, providing insights that complement existing research.

**Methods:**

A systematic literature search following the principles of a scoping review was conducted in the professional databases MEDLINE via PubMed and CINAHL via EBSCO in February and May 2022, as well as in August 2024. The characteristics of the included studies were summarised narratively in a pre-consented data extraction table. The synthesis of information was performed inductively and organised into three emergent themes: Career Entry, Role Development, and Retention.

**Results:**

31 publications from 13 countries were included in the synthesis and presentation of results. Among these, 18 studies examined factors inhibiting career entry, 10 studies explored role transition, and 3 studies investigated factors influencing retention. Barriers included insufficient team support, fear of making mistakes, and increased workload. Facilitators included support structures, such as mentors and peer networks, as well as designated transition periods.

**Conclusion:**

To promote the academic professionalisation of nursing in Germany and integrate of bachelor-qualified nurses into practice, structural frameworks embedded within the nursing process and oriented across multiple levels are essential. These frameworks should address both the barriers and facilitators identified in this review.

## Zusammenfassung

**Hintergrund:**

Bisherige Übersichtsarbeiten beleuchten einzelne Aspekte der frühen Berufslaufbahn Bachelor-qualifizierter Pflegefachpersonen – etwa Anpassungsstrategien, Herausforderungen im Arbeitsalltag oder Unterstützungsprogramme. Ein integrativer Überblick über das Zusammenspiel dieser Faktoren fehlt bislang.

Dieser Beitrag zeigt systematisch Treiber und Barrieren auf, die den Berufseinstieg, die Rollenentwicklung sowie den langfristigen Verbleib von Pflegefachpersonen mit primärqualifizierendem Hochschulabschluss beeinflussen.

**Methodik:**

Ein Scoping Review wurde nach den Richtlinien des Joanna Briggs Institute durchgeführt. Die systematische Literaturrecherche erfolgte in den Datenbanken MEDLINE (via PubMed) und CINAHL (via EBSCO) im Februar, Mai 2022 sowie im August 2024. Die Studienmerkmale wurden narrativ zusammengefasst. Die Analyse erfolgte induktiv und wurde thematisch in die Bereiche Berufseinstieg, Rollenentwicklung und Verbleib gegliedert.

**Ergebnisse:**

31 Studien aus 13 Ländern wurden eingeschlossen. Davon befassten sich 18 mit Faktoren zum Berufseinstieg, 10 mit der Rollenentwicklung und 3 mit dem Verbleib. Zentrale Barrieren waren mangelnde Teamunterstützung, Angst vor Fehlern und hohe Arbeitsbelastung. Förderlich wirkten sich die Unterstützung durch Mentor:innen, Peer-Netzwerke und strukturierte Übergangsphasen aus.

**Schlussfolgerung:**

Für die nachhaltige Integration von Bachelor-qualifizierter Pflegefachpersonen in der Pflegepraxis sind mehrstufige strukturelle Rahmenbedingungen erforderlich, die sowohl hemmende als auch förderliche Einflussfaktoren adressieren.

## Background

Nurses in Germany are qualified to provide independent, process-oriented care through either vocational training (a three-year apprenticeship) or university education (a three- to four-year bachelor’s degree) [https://www.buzer.de/9_Pflegeberufegesetz-PflBG.htm]. The regulation of nursing education at universities and universities of applied sciences was only introduced by the Nursing Profession Act in 2020. (The term “nurse” used in this paper corresponds to the term “registered nurse”, which is commonly used in many countries.) In 2022, the German Council of Science and Humanities reiterated its 2012 recommendation for 10 to 20% of nurses to be academically trained [51]. This stands in stark contrast to the actual proportion of university-qualified nurses, which was just 1.75% in 2021 [37]. In inpatient long-term care, their share of the total workforce is less than 1% [46]. Most bachelor-qualified nurses are employed in hospitals, often performing tasks similar to those of vocationally trained nurses, although some take on additional responsibilities or pursue advanced degrees [15].

The concepts of career entry, role transition, and retention are central to understanding the integration of bachelor-qualified nurses into professional practice. Career entry refers to the initial phase where newly qualified nurses adapt to the realities of clinical practice, navigate organisational structures, and begin applying their theoretical knowledge in real-world settings [5]. In contrast, role transition involves the developmental process of assuming independent professional responsibilities and establishing a sense of professional identity within a team and organizational context [16], [21]. While career entry focuses on mastering foundational competencies and adjusting to the workplace environment, role transition emphasises deeper integration into professional roles and managing interpersonal and organizational dynamics. Retention, meanwhile, encompasses strategies and factors that ensure the long-term sustainability of nurses in the workforce, including job satisfaction, career advancement opportunities, and institutional support [21].

This scoping review is embedded within a broader research project aimed at facilitating the successful and sustainable career entry of bachelor-qualified nurses. The project seeks to design tailored job profiles and develop concepts that healthcare and nursing organisations can use to systematically integrate bachelor-qualified nurses and create individualised career paths.

Existing theoretical frameworks provide valuable insights into distinct aspects of nurses’ career development. For instance, Duchscher’s transition framework emphasizes the emotional and social challenges faced during role transitions in the early stages of a nursing career. Baharum et al. [5] categorise factors influencing adaptation, such as work environments, personal attributes, and educational preparation. Hakvoort et al. [20] extend this perspective by exploring long-term factors that shape career development, such as continuing education and professional growth opportunities. However, these models primarily address individual phases or general nursing populations and do not integrate findings across the entire early career trajectory of bachelor-qualified nurses.

In addition, existing systematic reviews have addressed related aspects: Baharum et al. [5] identified adaptation strategies, Hawkins et al. [21] explored negative workplace behaviors, and Reebals et al. [43] examined support programs for new nurses. While valuable, these reviews lack a comprehensive perspective on the career trajectory of bachelor-qualified nurses across the phases of career entry, role transition, and retention.

This scoping review systematically identifies drivers and barriers across these three phases. By synthesising international findings and employing an inductive approach, it complements existing reviews and frameworks, offering actionable insights for healthcare systems integrating bachelor-qualified nurses, particularly in contexts like Germany, where academic nursing roles are still evolving.

## Objective

This scoping review aims to systematically explore and analyse the drivers and barriers influencing bachelor-qualified nurses during the early stages of their careers. While existing literature provides insights into topics such as workplace adaptation, negative workplace behaviors, and transition programs, it does not offer a comprehensive understanding of the specific challenges and opportunities encountered by this group.

Furthermore, the review complements the broader research project by supporting other surveys and laying the groundwork for developing strategies to effectively integrate university-educated nurses into the care sector.

## Method

The scoping review was conducted according to the methodological guidelines of the Johanna Briggs Institute [41] and reported according to the Preferred Reporting Items for Systematic Reviews and Meta-Analyses extension for Scoping Reviews (PRISMA-ScR) guidelines [50] . No protocol was developed a priori for this scoping review.

### Eligibility criteria

The inclusion and exclusion criteria for the selection of studies are shown in table 1 (Tab. 1). Only primary studies were included. Studies were included if they focused on identifying factors that influenced the implementation or integration process of bachelor-qualified nurses into health care practice, and studies that described influencing factors related to nursing management strategy. Only studies published between 2000 and 2024 in English or German with abstracts were included. Studies were excluded if they focused on nursing students, long-term staff or masters graduates. Studies focusing exclusively on Associate of Science in Nursing (ASN) graduates were also excluded. Book chapters, conference papers, and qualifying or doctoral theses were not included. Studies that did not focus exclusively on baccalaureate graduates were not excluded.

### Information sources

The literature databases MEDLINE via PubMed and CINAHL via EBSCO were searched systematically and independently by two persons (MZ & PL) in February, May 2022 and August 2024. In addition, the reference lists of the included full texts were searched for further references (JM). Grey literature was not included. 

### Search

The search term was developed (MZ), discussed and expanded by the entire research team, and the search terms adapted for each database are listed in the online supplement to this article (see attachment 1 ).

### Selection of sources of evidence

All articles were screened using the tool “rayyan” [24]. To increase consistency between reviewers, both authors (JM & PL) double-blindly reviewed the same publications for the specified inclusion and exclusion criteria and discussed the results. Both reviewers (JM & PL) assessed the titles, abstracts and full texts of all publications identified in the search for potentially relevant publications for consistency with the research question. 11 Conflicts were resolved by consensus and by the decision of a third reviewer (PJ).

### Data charting

A data charting form was developed jointly by two reviewers to determine which variables to extract. The two reviewers (JM & PL) independently charted the data, discussed the results, and continually updated the data charting form in an iterative process. 

### Data items

The characteristics of the studies were narratively summarised in a pre-prepared data table (JM) and included information on authors, year, study size and population, country of origin, study design, study objectives and results. The extracted results data were inductively analysed according to the specified focus (JM), i.e. the studies were not discussed, but structured thematically and reported as an evidence synthesis. The results of the analysis were reviewed and critically discussed by the research team.

## Results

### Selection of sources of evidence

**Figure 1:**
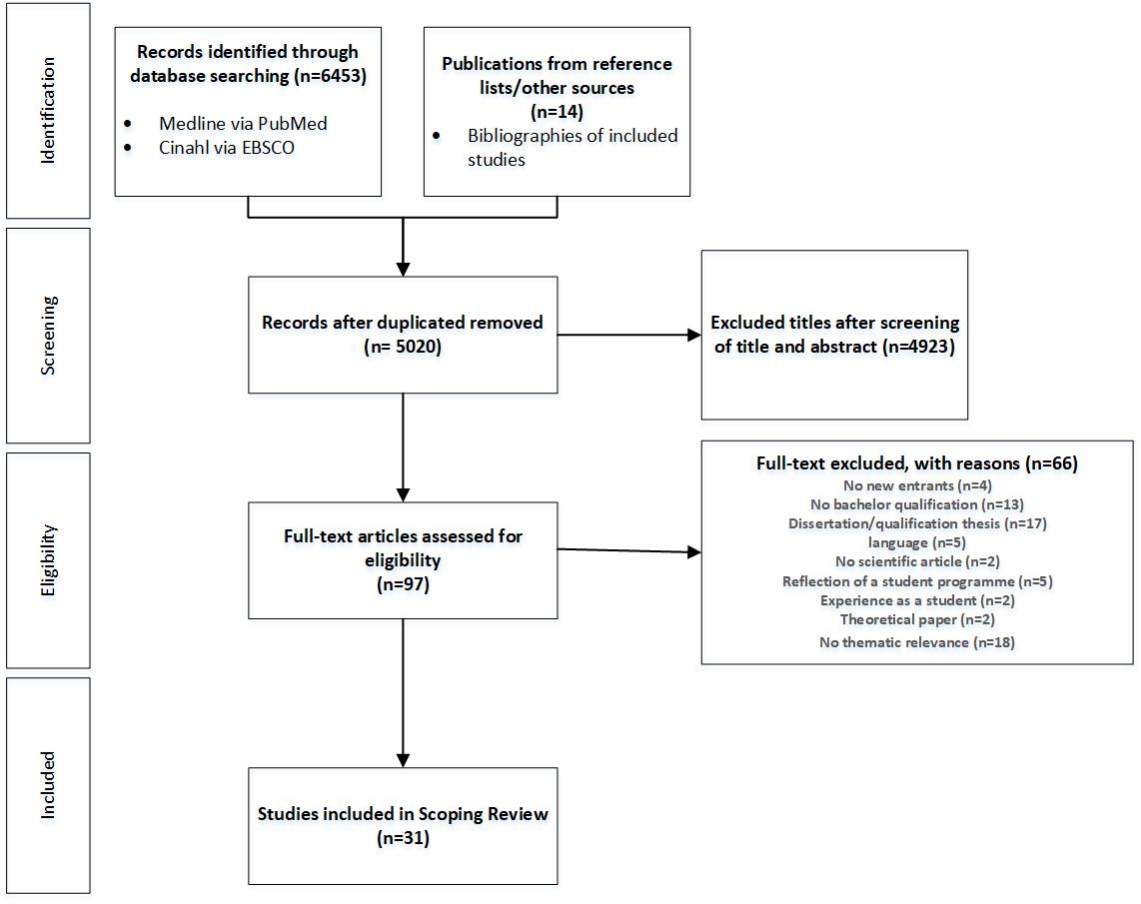
PRISMA flowchart for literature selection (Tricco et al. [50])

The systematic database search yielded 6453 studies, which were adjusted for 1433 duplicates. 5020 studies were reduced to 97 full texts after title and abstract screening. A further 14 studies were identified by screening the reference lists of eligible publications. After screening the 97 full texts for eligibility 31 studies were finally included. The literature search was conducted using the PRISMA ScR [50], see figure 1 (Fig. 1). 

### Characteristics of the included studies

All studies were written in English, except for one German paper. The studies were conducted in a total of 13 countries. Of these, three countries belonged to the European Union, which was represented by eight publications. There was one study from Germany. North America is represented by five studies from the USA and four publications from Canada. There are six studies from Australia, one from Africa and seven from Asia. Qualitative studies represent the majority, accounting for 25 studies, followed by three mixed-methods studies, one observational study, one observational study utilising multilevel statistical analysis and one quasi-experimental study. After reviewing the studies, they were grouped into three themes. Thus nine studies [6], [8], [10], [13], [25], [27], [35], [38], [44] identified factors that inhibit or promote career entry, nine studies [1], [12], [17], [22], [23], [26], [33], [36], [52] identified factors that inhibit or facilitate career entry in transition programmes, ten studies [9], [18], [19], [47], [48], [49], [32], [34], [39], [40] identified factors that inhibit or promote role transition from graduate to established nurse, three studies [7], [29], [30] and identified factors that inhibit or promote graduate retention and career development. 

A total of 2724 bachelor-qualified nurses were included in the 31 studies. In addition, care managers were included in two studies [17],[22], and experienced nurses in one study [22] and mentors in two other studies [12], [32].

The length of time a nurse was considered “newly qualified” varied between studies from less than 4 months to 2 years.

The characteristics of each study are listed in the online supplement (see attachment 2 ).

Attachment 3 provide an overview of the barriers and drivers identified in each study, categorised according to the three thematic areas. 

### Career entry 

Nine studies investigated the career entry of bachelor-qualified nurses [6], [8], [10], [13], [25], [27], [35], [38], [44].

Four studies identified factors that either facilitated or inhibited the transition of newly qualified Bachelor nurses [6], [13], [27], [35]. One study focused on the challenges faced by new nurses during this transition phase [25], while another explored the provision of transition support for early-career nurses [36]. Three studies delved into the experiences of newly qualified nurses as they navigated the transition process [10], [38], [44].

Three studies assessed the impact of transition programmes on bachelor-qualified nurses [12], [23], [26]. Two additional studies examined the challenges and opportunities associated with such programmes [1], [36].

Three studies identified strategies and key components for designing effective transition programmes [22], [33], [52]. Finally, one study analysed the learning processes of nurses participating in a transition programme, emphasising the importance of newly qualified Bachelor nurses recognising and embracing their professional roles [17].

### Role transition

Ten studies were categorised under the theme of role transition. 

Eight studies focused on the experiences of bachelor-qualified nurses transitioning from new nurse to professional practice [9], [18], [19], [48], [32], [34], [39], [40].

One study examined the needs of new graduates during role transitions [49], while another study investigated organisational stressors affecting professional commitment during this phase [47]. 

### Retention and career development 

Three studies were allocated to the theme of retention and career development. One study explored nurses’ motivations for leaving the nursing profession [30]. The authors interviewed 17 former nurses who had left the nursing profession after 2 years were interviewed. Another study identified factors that may contribute to sustainable retention of staff in hospital wards [29]. 

The only German study aimed to collect data on the career paths, job satisfaction and future prospects of academically trained nurses [7]. 77.6% of respondents reported that they were working in direct care. These respondents reported higher levels of dissatisfaction with their professional situation, with 81.3% seeing opportunities for further development. 31.1% of participants engaged in postgraduate study, with 39.6% were planning to do so. 

### Cross-theme insights

An analysis across the three themes revealed significant interconnectedness. Barriers such as lack of team support and high workloads frequently emerged across all phases, indicating that these challenges are not isolated to specific career stages but rather systemic issues. Similarly, enablers such as mentorship and structured feedback consistently facilitated positive outcomes throughout the career trajectory.

The cross-tabulation (see attachment 3 ) provides a comprehensive summary of these barriers and facilitators, demonstrating how they intersect across career entry, role transition, and retention.

## Discussion

As a foundational systematic synthesis of evidence, this scoping review analysed 31 studies investigating the barriers and drivers associated with integrating bachelor-qualified nurses into healthcare practice. The findings were organised into three emergent themes: “career entry”, “role transition”, and “retention and career progression”. For each theme, specific facilitators and potential interventions to address identified challenges were highlighted. Additionally, it was observed that the barriers and facilitators influencing the implementation of transition programmes closely resembled those identified in contexts without such programmes. To synthesise and clearly present the findings, a cross-tabulation was developed, providing a structured overview of the results (see attachment 3 ). 

In summary, organisations should prioritise good teamwork, collaboration with line managers, career prospects, and support services. While extensive international evidence exists in this area, future research should focus on the specific characteristics of the German context. In Germany, only 1.74% of a training cohort attains an academic nursing degree, compared to 45-100% in the countries analysed [37]. Despite long-standing recommendations [51], the importance of higher education frameworks for nursing remains under-recognised by policymakers. 

The benefits of employing academically qualified nurses – such as improved care quality and increased job and career satisfaction – are well documented (e.g., [3], [4], [11]. This satisfaction is closely linked to longer retention in the profession [2], [28], [31]. However, Bachelor programmes in Germany are still in their infancy and often fail to equip students with essential competencies such as advanced clinical decision-making, interprofessional collaboration, and leadership [14], [45]. A notable gap exists between the competencies taught in academic programmes and the expectations of clinical practice [14]. Additionally, the absence of clearly defined job profiles complicates the alignment of curricula with professional requirements [45]. 

International evidence, as reviewed in this scoping study, highlights the importance of structured clinical placements, mentoring systems, and training in soft skills such as teamwork and resilience to facilitate the transition from education to practice (see attachment 3 ).

This scoping review extends beyond existing systematic reviews by identifying critical similarities and differences between transition programmes and contexts without such programmes. The findings highlight that barriers such as insufficient team support, fear of making mistakes, and high workloads occur both with and without programmes [5], [20], [43]. Furthermore, the cross-tabulation developed in this scoping review systematically organises barriers and facilitators, highlighting the duality of factors such as mentorship and team support, which can act as both enablers and significant barriers when absent. This duality has not been comprehensively addressed in prior reviews and provides actionable insights for designing targeted interventions [5], [20].

The scoping review methodology provided a comprehensive overview of the research field. The database search string was developed systematically, using relevant keywords and search terms informed by the research objectives and preliminary literature analysis. This ensured the search strategy was robust and well-documented. The selection of studies was conducted in a double-blind manner, with conflicts resolved by a neutral reviewer (PJ), minimising potential bias.

Only one study conducted in Germany (2000-2024) was identified, raising questions about the transferability of the results. Given the relatively recent introduction of academic nursing degrees in Germany, a review of German grey literature could provide further insights. A systematic assessment of the validity of the included studies was not undertaken, as it was beyond the scope of this review.

## Conclusion and implications for practice

The existing research on the integration of bachelor-qualified nurses offers a wide range of international findings. However, from a German perspective, the predominance of international studies requires critical evaluation due to differences in healthcare systems, educational structures, and professional expectations. For instance, the lack of standardised national guidelines for nursing curricula and varying employer expectations in Germany may present unique barriers.

Future research should explore these specific challenges in the German context. Key questions include:

- How do employer and team expectations differ from the competencies taught in bachelor programmes?
- To what extent do continuing education and career development programmes impact job satisfaction and retention?
- What support measures (e.g., mentoring, transition programmes) can ease the transition from education to practice?
- How do experiences of bachelor-qualified nurses differ across care settings (e.g., hospitals, long-term care)?

Addressing these questions requires methodological diversity, combining qualitative stakeholder insights (e.g., focus groups, interviews) with quantitative data (e.g., surveys, workforce analytics). This mixed-methods approach provides a comprehensive understanding of Germany’s unique challenges.

This review highlights the need to develop and evaluate implementation concepts for integrating Bachelor-qualified nurses into Germany’s healthcare system. These efforts should include pilot projects, longitudinal studies, and comparative analyses to identify best practices and measure outcomes.

In summary, targeted and methodologically diverse research is needed to bridge the gap between international findings and Germany’s specific requirements, facilitating the effective integration of Bachelor-qualified nurses.

## Notes

### Author contributions

All authors have agreed on the final version and fulfil at least one of the following criteria (according to ICMJE [https://www.icmje.org/recommendations/]):

- Significant contributions to conception and design, data collection or data ana-lysis and interpretation.
- Article writing or critical revision for important intellectual content.

The authors Denny Paulicke and Patrick Jahn share the last authorship.

### Authors’ ORCIDs

- Julia Müller: [0009-0003-6395-168X]
- Florian Breitinger: [0000-0001-8750-6271]
- Patricia Bräuer: [0000-0003-2979-824X]
- Bettina Dauer: [0000-0002-9436-2605]
- Max Zilezinski: [0000-0001-9225-1672]
- Denny Paulicke: [0000-0002-9808-215X]
- Patrick Jahn: [0000-0002-1533-6717]

### Funding

This study was funded by the Federal Institute for Vocational Education and Training (BIBB) from contract research funds. Order number: 9510160237.

### Approval by the Ethics Committee

Advice from the Ethics Committee was obtained. No ethics committee approval is required due to the study design.

### Availability of data and materials

All studies and materials analysed during this research are included in this published article and its supplementary appendices.

## Acknowledgements

We would like to thank the participating institutions and other project collaborators for their cooperation. We would also like to thank Dr. Anne-Marie Lachmund who was responsible for proof-reading and stylistic adjustments. 

## Competing interests

The authors declare that they have no competing interests. 

## Supplementary Material

Search strings of the literature search in the individual databases

Characteristics and results of the individual studies

Cross-classification of barriers and drivers in the areas of careed entry, role transition and retention

## Figures and Tables

**Table 1:**
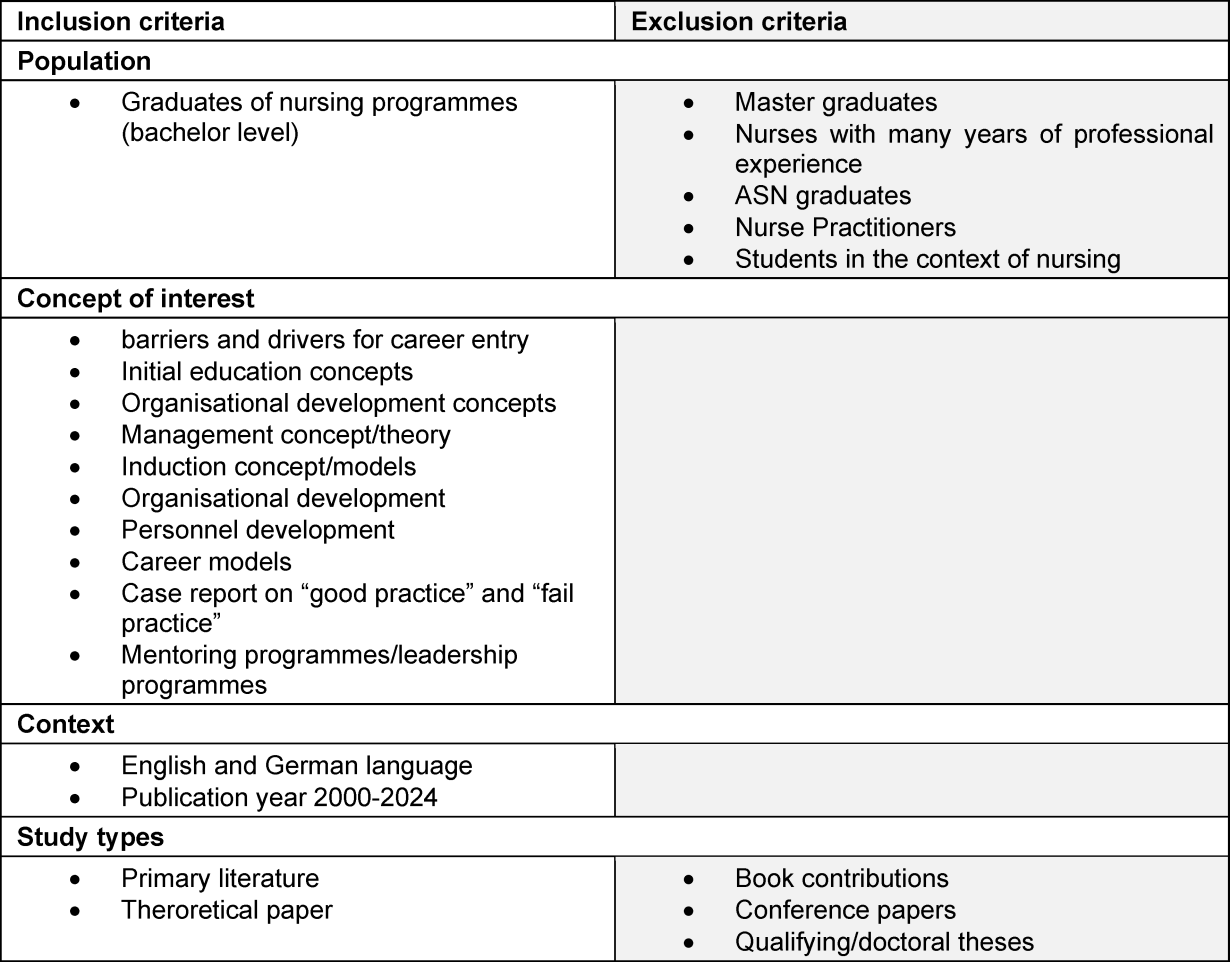
Inclusion and exclusion criteria
