# Supplementary material for: Drivers and barriers to career entry and retention of nurses with initial higher education: A scoping review
Source: GMS J Med Educ. 2025 Jun 16;42(3):Doc35. doi: 10.3205/zma001759 (PMC12286869; doi:10.3205/zma001759)
Supplement: Search strings of the literature search in the individual databases [file JME-42-35-s-001.pdf]

Attachment 1: Search strings of the literature search in the individual databases

**PubMed via Medline**	(("baccalaureate"[All Fields] OR "baccalaureates"[All Fields] OR ("bachelor"[All Fields] OR "bachelor s"[All Fields] OR "bachelors"[All Fields]) OR ("novice"[All Fields] OR "novice s"[All Fields] OR "novices"[All Fields])) AND "nurs*"[All Fields])<br>OR<br>("clinical competenc*"[All Fields] OR "nurses role"[All Fields] OR "professional role"[All Fields] OR "professional*"[All Fields] OR “clinical practice”[All Fields])<br>AND<br>("barrier"[All Fields] OR "barrier s"[All Fields] OR "barriers"[All Fields] OR "hurdle"[All Fields] OR "hurdles"[All Fields] OR "facilitate"[All Fields] OR "facilitated"[All Fields] OR "facilitates"[All Fields] OR "facilitating"[All Fields] OR "facilitation"[All Fields] OR "facilitations"[All Fields] OR "facilitative"[All Fields] OR "facilitator"[All Fields] OR "facilitator s"[All Fields] OR "facilitators"[All Fields]] OR "implement*"[All Fields])<br>AND<br>("Management concept"[All Fields] OR "Organisational development"[All Fields] OR "inducation"[All Fields] OR "Personnel development"[All Fields] OR "Career models"[All Fields] OR "leadership"[MeSH Terms] OR "leadership"[All Fields] OR "leadership s"[All Fields] OR "leaderships"[All Fields] OR "transit"[All Fields] OR "transited"[All Fields] OR "transiting"[All Fields] OR "transition"[All Fields] OR "transitional"[All Fields] OR "transitionals"[All Fields] OR "transitioned"[All Fields] OR "transitioning"[All Fields] OR "transitions"[All Fields] OR "transits"[All Fields]) OR "nursing management"[All Fields] OR "personnel management"[All Fields] OR "professional engagement"[All Fields] OR "vocational adjustment"[All Fields] AND ("theorie"[All Fields] OR "theories"[All Fields] OR "theory"[All Fields] OR "theory s"[All Fields])
**Cinahl via EBSCO**	"TI (nurs or nurse or nursing or nurses) AND AB (Baccalaureate OR Bachelor OR Novice) OR AB ("clinical nurse specialist*" OR "nurse specialist" OR "clinical competenc*" OR OR "nurses role" OR “professional role” OR professional* OR “clinical practice”) AND TI (barriers OR hurdles OR facilitators OR implement*) AND AB ("management concept" OR "organisational development" OR inducation OR "personnel development" OR "career models" OR leadership" OR transition OR “nursing management” OR "personnel management" OR "professional engagement” OR "vocational adjustment" AND theory)

Attachment 1 to Müller J, Breitinger F, Bräuer P, Dauer B, Zilenzinski M, Paulicke D, Jahn P. *Drivers and barriers to career entry and retention of nurses with initial higher education: a scoping review*. GMS J Med Educ. 2025;42(3):Doc35. DOI: 10.3205/zma001759
