# Supplementary material for: Drivers and barriers to career entry and retention of nurses with initial higher education: A scoping review
Source: GMS J Med Educ. 2025 Jun 16;42(3):Doc35. doi: 10.3205/zma001759 (PMC12286869; doi:10.3205/zma001759)
Supplement: Characteristics and results of the individual studies [file JME-42-35-s-002.pdf]

Attachment 2: Characteristics and results of the individual studies

Themes	Authors/ year	Study size & population	Country of origin	Research design	Study objectives	Results
Career entry	(Kim und Shin 2020)	In quantitative phase (questionnaire survey): 230 graduates with a university degree.<br>In the qualitative phase: 22 young professionals in three focus groups	Korea	Mixed-methods-design	Identifying factors that hinder or facilitate the successful transition of new carers	Quantitative results:<br>• emergency situations, prioritisation/time management, and respiratory, death and end-of-life care were the most difficult skills for entry-level nurses.<br>• multiple regression analysis revealed most important factors for transition:<br>o self-efficacy (β = 0.27, $p < .01$),<br>o job satisfaction (β = 0.27, $p < .01$),<br>o job stress (β = -0.04, $p < .05$)<br>o structural agency (β = 0.41, $p < .01$)<br><br>Qualitative results:<br>• barriers<br>o fear of making mistakes, communication with doctors and senior nurses, dying patients<br>o expectations of other nurses: caring for the same number of patients as experienced nurses.<br>o excessive workload<br>o excessive role expectations → exuding competence as an expert vs. to behave like a newcomer to the profession<br>o emotional stress due to bullying → reporting of mistakes by novice by others<br>• drivers<br>o increased self-confidence through experiences of success<br>o stress reduction through interaction with colleagues in the same ward<br>o positive, supportive work environment through feedback from mentors<br>o understanding of making mistakes by senior nurses

						o need for a continuous transition programme for skills and knowledge training as well as strategy training (stress reduction, time management, interaction/communication in the hospital) → six months to one year
Career entry	(Baldwin et al. 2021)	26 new nurses (BSN, ADN, Diploma) in specialised areas in the hospital (Emergency Department, (Paediatric ICU, IMC, OP).	USA	Qualitative, descriptive study	Investigating how new nurses adapt to the expectations of high severity specialty areas<br>Detailed objectives:<br>1. describe personal factors that facilitate or hinder a successful transition of new nurses into the specialty areas<br>2. description of factors in the specialty area that facilitate or hinder successful transition of new nurses<br>3. identification of recommendations, suggestions or ideas from the subjects or ideas that might facilitate the transition for future new nurses; and<br>4. comparison of the respondent's experiences in one specialty area with other new nurses on other wards	Drivers and barriers<br>Importance of ward culture → 3 subtopics: communication style, teamwork, degree of integrati-on.<br>Drivers:<br>o new nurses valued a positive, supportive, professional style of communication, especially with mentors, men-tors who took their time, welcomed them well, advocated for them and offered explanations.<br>o drivers: good cooperation for safe patient care and integration of new nurses.<br>Barriers:<br>o impatient mentors and unrealistic expectations;<br>o interactions with non-team-oriented, experienced nurses and rudeness<br>o lack of materials and support staff → Time management problems<br>o delays in care<br>o learning disabilities due to lack of staff, too few patients on the ward strategies<br><br>Identification of two main categories:<br>(a) individual strategies:<br>• Talking to a person about their experience who understands the situation;<br>• seek help to avoid mistakes<br>• being open to feedback as well as self-critical assessment of one's own performance

						(b) Institutional strategies:<br>• Better coordination between implementation programme staff and new nurses to promote new nurses to practice.<br>• Use of good mentors who provide support and build confidence<br>• Friendly behaviour of staff and mentors for good working culture<br>• Recommendation for additional courses with specialised knowledge,<br>• more practice in the implementation programme;<br>• more practice sessions with more tools<br>• Introduction of complicated electronic medical record too early in the programme
Career entry	(Bisholt 2012a)	18 new nurses in a rural hospital in Sweden	Sweden	Ethnographic study (participant observation, interviews and field notes)	Analysis and description of how newly qualified nurses learn on the job and how they seek meaning in practice.<br><br>What is the meaning of the induction programme experience for new nurses?<br>What obstacles and opportunities do newly qualified nurses experience in their interactions with supervisors and other staff?<br>What strategies do newly qualified nurses develop to learn on the job?	Knowledge acquisition of new nurses through interaction with experienced nurses, emergence of a master-apprentice system to practice mainly technical and practical tasks.<br><br>Barriers/opportunities:<br>• little experience with complex acute patient situations<br>• recommendation no night work for new nurses without experienced nurses<br>• too little reflection of new nurses in care situations<br>• tasks are carried out according to the instructions of the experienced nurses<br>• new nurses do not reflect on the actions of experienced nurses in terms of correctness.<br>• acceptance in the team is essential for new nurses

Career entry	(Dames 2019)	Eight new nurses (six months to two years experience) Recruitment via third party website	Canada	Qualitative study	Investigating the impact of the interplay of workplace and developmental factors that promote or hinder the development of new nurses	• intensity of stress varied from the degree of familiarity in the environment as well as the acceptance into the nursing team.<br>• self-esteem problems → high level of tension and stress<br>• poor rest and stress due to shift work<br>• introversion, anxiety and depression in previous history increased stress, lack of sleep increased feelings of stress<br>• feeling of pressure to keep up with experienced colleagues<br>• too much workload in care with little experience, with increasing experience → meeting needs<br>• sense of belonging and acceptance by colleagues makes it easier to ask for help<br>• threats and physical violence by patients lead to (chronic) stress<br>• lack of conflict resolution in the team, colleagues talking behind each other's backs, excessive reporting of mistakes by newcomers to the profession<br>• no freedom of expression possible, lack of recognition<br>• mentorship encourages commitment to self-fulfilment<br>• lack of mentorship leads to chronic stress and sick leave<br>• self-compassion supports a more optimistic attitude
Career entry	(Brandt et al. 2017)	Seven new nurses with twelve to 15 months work experience	USA	interpretative, descriptive Survey	Survey of new nurses' experiences of transition to practice in the first twelve months after graduation	• orientation programmes facilitate transition to practice<br>• intense situations trigger strong emotions<br>• patient safety is the main concern<br>• fears of being on one's own → mentoring support essential → contributes to bonding<br>• stress decreases with experience

Career entry	(Maddalena et al. 2012)	Ten new nurses (degree unknown)	Canada	Qualitative study	Investigating the factors that influence the quality of the working life of new nurses	High motivation of new nurses to provide high quality patient care, despite many stressors:<br>• "difficult personalities"<br>• inadequate induction and supervision<br>• horizontal violence by nursing and medical colleagues<br>intensification of stress factors due to<br>• shortage of staff<br>• high workload<br>Key factors for a successful transition:<br>• supportive mentoring<br>• appropriate induction
Career entry	(Hu et al. 2017)	25 new nurses in paediatrics	China	Qualitative study	Investigation into the stress factors of new nurses in paediatrics in a Shanghai hospital	Identification of numerous stress factors:<br>• low work status<br>• insufficient professional competence<br>• high workload<br>• inadequate support systems; and<br>• uncertainty about career development
Career entry	(Nour und Williams 2019)	14 new nursing professionals who work in acute care.	Canada	Qualitative study (Grounded theory)	Examining the experiences of new nurses in acute health care settings in Canada	• young professionals are not ready to work independently after graduation<br>• expressed overwhelm and frustration at the beginning<br>• induction and learning under supervision did not meet expectations → lack of support, lack of availability of mentors<br>• increase in knowledge with experience<br>• driver: development of self-confidence
Career entry	(Regan et al. 2017)	42 new nurses and 28 care managers from seven Canadian regions	Canada	Descriptive, qualitative study	Examining the transition experience in Canadian health care through the perspective of new nurses and nurse managers in individual departments	Facilitating factors in the transition to practice were named by the new nursing professionals and the nursing service managers alike:<br>• formal orientation programmes<br>• ward culture<br>• promotion of constructive feedback and supportive mentors.

						barriers:<br>• unforeseen changes in the duration of orientation<br>• inadequate staffing<br>• rude ward culture<br>• high workload
Career entry with transition pro-grammes	(Mellor und Greenhill 2014)	21 entrants to the profession who had completed their transition to practice programme.	Australia	Qualitative design with group interviews (grounded theory)	Exploring the nature of support within transition programs for new nurses in rural areas	Three key themes were identified:<br>• too little preparation for practice → expectations to the role of nurse quite different from the student; difficulty in prioritising care, during placement focus on single tasks and ideal number of patients; no opportunity to take on more patients as well as other aspects of the role of a nurse<br>• overwork and being left alone → lack of induction, new nurses have to work alone, system deficiencies e.g., unreliable on-call procedure, no role fulfilment by mentors, lack of feedback, no services with practice supervisor/ no debriefing, absences in interprofessional context<br>• need for clinical supervision → interest in evidence-based practice and guidance in developing their role as a professional nurse, concern about situations requiring experience, concern about making gross errors in practice, interest in working with experienced colleagues, frustration as this did not happen
Career entry with transition pro-grammes	(Eklund et al. 2021)	31 nurses with a bachelor's degree with less than four months of work experience and 8 ward managers in 6 hospitals	Sweden	Qualitative design with group interviews	Investigating the learning processes of newly graduated nurses in a 1-year transition programme	To identify three themes that emerged as important in the learning process during the transition programme. Professional knowledge developed through supporting these processes<br>• recognition of the role of the new nurse as an entrant to the profession (includes personal understanding of lack of specific knowledge after graduation, understanding of the role as an independent nurse, separate from being a student → great influence through support of colleagues and supervisors → sharpens self-reflection of the entrants as well as their commitment; fulfilling a double role: fulfilling tasks but also sufficient sense of security in the work group)

						• emphasising the role of the new nurse as a learner (need for a structured introduction at the beginning of the programme, which according to the ward managers had been postponed due to the high workload; strength of the programme: combination of daily work, training days and reflection opportunities)<br>development towards a comprehensive role in nursing (strength of the programme, according to the entrants, was the combination of reflection seminars with other entrants on professional practice, lectures and personal practice to develop the ability to provide highly complex patient care)
Career entry with transition pro-grammes	(Henderson et al. 2015)	78 BSN graduates twelve months after starting work in a hospital	Australia	Mixed-methods-design (survey + focus groups)	Investigating the benefits of the workplace engagement support programme for new nurses	Survey:<br>• positive response to transition programme<br>• important components in the workplace: sense of belonging, recognition and realisation,<br>• graduates reported no influence on changes in practice<br><br>Focus groups:<br>Three core aspects were essential for transition to practice:<br>(1) the importance of study days, especially networking during events.<br>(2) positive working relationships with mentors<br>(3) the positive contribution of the care team.
Career entry with transition pro-grammes	(Hayton et al. 2021)	Six newly qualified nurses (focus group interview), 28 newly qualified nurses (qualitative questionnaire), 17 experienced nurses (qualitative questionnaire);	South Africa	Qualitative, interpretative, descriptive study	Establishing a conceptual framework for a person-centred transition programme	Conceptual framework includes four areas:<br>• learning outcomes (application of learning in practice, development of problem solving skills, conflict management in the workplace, ward management, well-being (through respect, appreciation and support), professional socialisation (through adopting values, healthy relationships and person-centred care)).<br>• learning processes (interprofessional cooperation is a prerequisite),<br>• learning environment (developing a sense of security),

		four studies on the experiences and competencies of newly qualified nurses in South Africa				caregiver prerequisites (professionally competent, confident, at least three years of experience, have effective time management skills)
Career entry with transition pro-grammes	(AbuAlRub und Abu Alhaija'a 2019)	30 new nurses in their first year in three hospitals	Jordan	Qualitative descriptive study	Investigation on the challenges of Jordanian nurses in the first year and the opportunities and challenges of implementing an induction programme	• lack of national familiarisation programmes<br>• discrepancy between theory and practice<br>• lack of role models → experienced nurses leave the country<br>• lack of self-confidence due to lack of skills<br>• Reality shock after graduation, usually no familiarisation programmes in hospitals, high stress → Burnout → planning to leave the workplace<br>• too little practical relevance in studies
Career entry with transition pro-grammes	(Yao et al. 2021)	154 new nurses (associate degree and BSN)	China	Quasi-experimental study	Developing a standardised training programme for newly graduated nurses.<br><br>Studying the implementation and evaluating the impact of the programme, and collecting the opinions of the participating nurses	• Standardised training programme consisted of theoretical instruction and practical training (duration six months) and was based on core competencies of nurses.<br>• participation in 69 hours of classroom and four months of clinical practice training<br>• Course instructors from three high-level psychiatric hospitals → senior nurses, professors<br>• development of a clinical practice training manual for newly graduated nurses<br>• continuous guidance during the four months of clinical practice training by experienced mentors trained before the start of clinical practice<br>• conducting a case-based nursing assessment every 2 weeks followed by mentor evaluation<br><br>• improvement of participants' core competencies as well as support for professional development<br>• recommendation: extension of the training period

Career entry with transition pro-grammes	(Lima et al. 2016)	21 new nurses who participated in a twelve-month transition programme	Australia	Qualitative explanatory method as part of a mixed methods design	Developing a framework for competence development of new nurses in paediatric nursing in a transitional programme	The findings from the interviews, combined with the preliminary model and the quantitative results, enabled the conceptualisation of a framework for developing competence<br>Key elements for competence development<br>• the individual in the team<br>o support from team → greater competence development<br>o little support from team → lower competence development<br>o educating nurses → great importance<br>o competence development by others: doctors, patients (difficult to talk to both parties) and their families (source of information)<br>• identification and interpretation of standards<br>o clinical standards support competence development<br>o non-clinical standards were not addressed<br>• asking questions<br>o competence development through "asking questions<br>o perceived barriers by educating nurses → reference clinical guidelines<br>o competence development through being asked questions<br>• guidance and engagement<br>o improving competence through feedback, support, challenge<br>o competence development from the interplay of positive and negative feedback<br>o familiarisation time and overtime<br>o mitigating overload by working with the mentor<br>o competence development through observing students in activities<br>o continuous professional development through training as well as self-study

Career entry with transition pro-grammes	(Hussein et al. 2017)	87 new nurses	Australia	Survey of a mixed methods design	Investigation of changes in perceptions of graduate nurses during the twelve-month transition support programme.<br><br>Identifying organisational factors and elements of clinical care that influenced their experience	Quantitative results:<br>• on two measurement points → no significant differences in satisfaction with the clinical practice environment (mean PES-AUS, scores: 112.4 versus 110.7, p = 0.298); ii) overall satisfaction with the transition support programme (mean: 7. 6 versus 7.8, p = 0.337); iii) satisfaction with the number of training days (mean: 4.4 versus 4.7, p = 0.72); iv) orientation days received (mean: 6.4 versus 6.6, p = 0.541); v) ward orientation (mean: 4.4 versus 4.8, p = 0.081); vi) confidence level (mean: 3.6 versus 3.5, p = 0.933); and vii) not practicing beyond one's clinical skills (mean: 3.9 versus 4.0, p = 0.629), across the two time periods.<br><br>Qualitative results:<br>• induction and transition programme essential for transition<br>o good emotional and clinical support from the team.<br>o additional support from clinical nurse educators, ward managers, nurses → essential for acceptance and promotion of learning<br>o some experienced staff → unrealistic expectations of clinical skills, others took little time for induction<br>o new staff not always introduced to the team<br>• transition programme provided opportunity to develop clinical skills<br>o access varied according to availability and expertise of transition programme coordinator, nurses, team leaders, senior managers<br>o barriers: increasing workload, inadequate nursing skill mix, unexpected clinical situations such as emergency medical team calls, dealing with aggressive patients, patients with poor general health, challenging families

Career entry with transition pro-grammes	(Cox et al. 2023)	93 nurse participants and 62 mentors	Australia	Mixed-Methods-Design	Determining the impact of the Transition to Practice Program (TPP) on participants' primary health care nursing knowledge, skills, and confidence, and their overall satisfaction with the program	The 12-month TPP comprises<br>• a 1-day face-to-face induction workshop,<br>• the provision of clinical and professional mentoring,<br>• 12 core modules based on the agreed PHC education topics,<br>• a self-assessment framework that participants used to benchmark their progress and identify learning goals<br>• In addition to these components, informal support was provided by Australian Primary Health Care Nurses Association (APNA) via teleconference.<br>• Service agreements with participants' workplaces were established to enable protected time (57 h over the 12-month program)<br><br>Drivers<br><br>• TPP was relevant and effective for nurses transitioning to PHC<br>• clinical and professional mentoring as a key enabling factor in this program<br>• significant increases in knowledge, skills, and confidence about working in PHC<br><br>barriers<br>• finding suitable mentors for all participants was challenging
Role transition	(Bisholt 2012b)	18 new nurses in a rural hospital in Sweden	Sweden	Ethnography study (participant observations, interviews, and field notes)	Description and analysis of how new nurses are socialised into the profession	Identification of five key themes:<br>• "being accepted in the profession"→ colleagues reinforced and trusted professional skills of novice nurses.<br>• "being questioned in the profession" → questioning of skills by other staff, increase in insecurities of new nurses as well as own demands for professionalism<br>• "being integrated into a hierarchical organization" → other staff members emphasized their superior

						position to the new nursing professional through sarcasm, laughter, or exasperation,<br>• „finding oneself a stranger" → excluding the new nurse by ignoring him/her, blaming him/her, not offering support, talking to the boss about new nurse<br>• „developing by taking on responsibility“ → decision by doctors, other nurses and support staff whether new nurse should be allowed to take on tasks
Role transition	(Hoeve et al. 2020)	580 diary entries from 18 new nursing professionals	Netherlands	Cohort observation design	Examination of the most important organisational work stressors to new nurses´ professional engagement, examination of whether work stressors are mediated by negative emotions	Most negatively associated with professional commitment:<br>• lack of support from colleagues,<br>• negative experiences with patients, and<br>• confrontation with existential events.<br><br>Other organisational work stressors indirectly and negatively associated with engagement:<br>• complexity of care<br>• lack of control<br>• imbalance between work and personal life<br>• directly related to job engagement were:<br>• conflicting work demands<br>• lack of control
Role transition	(Feng und Tsai 2012)	Seven new nurses from four medical centres in Taiwan	Taiwan	Qualitative, descriptive study	Investigation into the socialisation experiences of new nurses (BSN)	Identification of three themes:<br>• overwhelmed chaos → unclear roles, ignorance, inexperience, new skills, uncertainty in dealing with mistakes (Who helps? What to do?), overwhelm, lack of staff, lack of support, not enough responsibility as students<br>• learning by Doing → knowledge from studies too general, learning on own, advanced trainings were not perceived: too difficult, outside working hours, too exhausted

						• being an insider → after five months less professional stress, feeling of being part of the team seemed important, at the beginning compulsion to comply and adapt in order not to be yelled at, keeping knowledge constantly up to date, adjustments to gap between professional value (patient-oriented) and organisational value (task-oriented)
Role transition	(Paech 2002)	Eight new nurses	Australia	Qualitative study (pheno-menology)	Investigation into nurses´ experiences of role transition from student to new nurse	Identification of three themes:<br>• responsibility and accountability → discord between too much responsibility and new freedom, partly no induction to new role<br>• changes → fears in transition, good support from friends and partners, colleagues felt threatened by new nurses, exclusion, felt more comfortable in areas they already knew, previous experience is important<br>• job satisfaction → self-esteem boosts self-confidence, realisation of a dream, financial gain, more opportunities
Role transition	(Hoeve et al. 2018a)	18 new nursing professionals documenting their work-related experiences in 18 diaries (580 diary entries)	Nether-lands	Multilevel study	Investigation into the contextual, relational, and cognitive factors derived from the work experiences of novice nurses that affect emotions and affective commitment	• negative relationship between complexity of care, lack of support, and lack of competence with affective commitment of new nurses<br>• positive correlation between support received and affective commitment
Role transition	(Ortiz 2016)	Twelve new nurses (associate degree, bachelor´s degree) who have been working at the hospital in	USA	Descriptive, qualitative study	Examination of new nurses' description of their lack of self-confidence when entering professional practice and the development of self-confidence in the first year of practice in hospital	Identification of key issues on reasons for lack of confidence:<br>• communication is important,<br>○ bad experiences in communicating with doctors, patients, mentors → Negative comments reduce professional self-confidence<br>• making mistakes<br>○ medication errors, patient falls

		New York for up to one year.				• discrepancy between theory and practice<br>• poor preparation for caring for more than one patient at a time, dealing with angry doctors or family members<br><br>Identification of key issues to develop confidence:<br>• building relationships<br>o with doctors, colleagues, mentors<br>• independence<br>o making independent decisions<br>• positive feedback is important<br>o from patients and mentors<br>• gathering experience
Role transition	(Lyman et al. 2020)	13 new entrants (in the last two years) working in hospital wards	USA	Descriptive, qualitative study	Investigating the experiences of new nurses with mental safety	Identification of four main issues that play an overriding role in the mental safety of young professionals<br>• building credibility<br>o developing confidence in one's own nursing practice and that of others;<br>o strengthening credibility by gaining clinical experience and demonstrating appropriate knowledge, judgement and skills.<br>o gossip and condescending words weaken recognition<br>o interest and asking questions eliminated mentors' concerns<br>• feeling supported<br>o helped by the team, managers and clinic<br>o felt supported when they were "heard"<br>o those who did not feel supported are more anxious and less satisfied with their work<br>• building relationships<br>o relationships that go beyond the workplace → reducing anxiety

						• seeking security<br>o in choosing a job<br>o engaging with groups/people who make them feel safe (asking questions)<br>o avoiding unsafe situations through silence or workarounds e.g. little contact with carers who make them feel unsafe
Role transition	(Hoeve et al. 2018b)	18 new nurses (BSN) in a university hospital in the Netherlands	Nether-lands	Qualitative longitudinal study	Exploring the personal and professional needs of new nurses and factors to improve the transition from novice to professional nurse	Identification of eight key themes:<br>• relationships: most reported; more negative experiences with patients than positive, support from team and mentors mostly positive, relationship experience with doctors mostly negative<br>• experiences with competence mostly positive: applying knowledge and skills, causing mistakes, perception of own competence<br>• development: (lack of) opportunities for further development, experience in organisational context mostly negative<br>• impairment of well-being: Complex care of patients, work pressure, physically and cognitively stressful work<br>• existential events: Contact with dying, very ill, young patients very stressful; confrontation with feelings of guilt,<br>• goals: professional/private future plans, work-life balance negatively burdened<br>• autonomy: feeling of control/loss of control decisive for professional development<br>• identification: mostly professional satisfaction with regard to career choice
Role transition	(Leong und Crossman 2015)	26 new nurses and 31 mentors in five different hospitals.	Singapore	Qualitative study (Grounded Theory)	Investigating new nurses' perceptions of their experience of role change and the implications for leaders in terms of training, development and retention	• induction took place through an induction programme<br>• new nurses had a mentor but had to meet different expectations, continuous same service with mentor not possible → service with elders<br>• staff shortage<br>• mentor-to-newcomer ratio (1:8) too high<br>• little intrinsic motivation of mentors to teach

						• learning impediments due to negative workplace cultures (arbitrary management behaviour influences intention to leave workplace)<br>• preliminary internship facilitates transition to practice<br>• little motivation on the part of ward managers to train students who do not return after training<br>• stress due to ignorance and pressure to comply and adopt routines → leads to resignation<br>• not showing emotions at the workplace<br>• facilitating induction through observation, questioning and social support<br>• driver: Willingness to learn
Role transition	(Gautam et al. 2023)	10 nursing graduates (BSc. Nursing) from two private hospitals in Nepal (Work experience 4-12 months)	Nepal	Qualitative decriptive study	Exploring new nursing graduates' perceptions of the transition to professional practice.	Identification of four key themes that encompassed new nursing graduates' transition<br>Experiences:<br>• "getting hit by reality"<br>○ High level of work that requires minimal theoretical knowledge<br>○ Large gap between theory and practice<br>○ Lack of support after graduation<br>○ Little responsibility during the degree programme, high level of tasks after graduation<br>• "losing confidence"<br>○ Great sense of anxiety about taking on major tasks<br>○ Fear of making mistakes, including a growing need for validation from superiors<br>○ lack of self-confidence, working according to the instructions of superiors<br>○ Ignorance from patients and visitors as they were initially unable to answer questions<br>○ Blame from senior colleagues

						• "feeling unsupported"<br>○ Lack of familiarisation and training, resulting in errors at work<br>○ Lack of support from senior colleagues<br>• "gathering strengths"<br>○ Learning independently by reading books or watching videos<br>○ Asking senior colleagues for help
Retention and pro-fessional develop-ment	(Kovner et al. 2016)	1335 nursing graduates from large cities and rural areas in the hospital setting (all degrees)	USA	Panel survey	Identifying factors that predict retention of new nurses in a department, position, to develop retention strategies at department level	Identification of five variables with the greatest impact on retention in a department:<br>• variety (positive)<br>• another job for pay (negative)<br>• first undergraduate degree (a bachelor's or higher degree increased the likelihood of retention)<br>• negative affectivity (positive)<br>• job satisfaction (positive)
Retention and pro-fessional develop-ment	(Kox et al. 2020)	17 former nurses who left the profession two years after graduation	Nether-lands	Qualitative study	Investigating the motives of Dutch nurses to leave the nursing profession	Identification of six key issues:<br>• lack of challenge; ambition to progress in management or research positions.<br>• lack of passion; no sense of passion for patient care.<br>• lack of perceived competence; feeling "not ready to take challenge"<br>• lack of job satisfaction due to high workload; work-life imbalance and inability to provide quality care.<br>• lack of ability to work due to non-occupational health conditions; failure to meet requirements for adaptation to the workplace or work environment<br>• lack of sense of belonging; negative attitude of colleagues towards each other
Retention and pro-fessional develop-ment	(Baumann und Kugler 2019)	273 Bachelor graduates from undergraduate qualifying nursing degree courses	Germany	Online survey in cross-sectional design	Collection of data on the career, occupational satisfaction and future prospects of graduates of nursing programmes	• 77.6% of respondents → employment in health care and nursing/elderly care → higher dissatisfaction with professional situation than respondents in extended practice fields<br>• there is a high degree of satisfaction with regard to qualification-related scope for action and general conditions.

						• in terms of professional development, 81.3% of those surveyed see opportunities for further development.<br>• 31.1% of respondents are currently pursuing further studies and 39.6% plan to take up such studies in the future
