# Supplementary material for: Drivers and barriers to career entry and retention of nurses with initial higher education: A scoping review
Source: GMS J Med Educ. 2025 Jun 16;42(3):Doc35. doi: 10.3205/zma001759 (PMC12286869; doi:10.3205/zma001759)
Supplement: Cross-classification of barriers and drivers in the areas of careed entry, role transition and retention [file JME-42-35-s-003.pdf]

Attachment 3: Cross-classification of barriers and drivers in the areas of careed entry, role transition and retention

Barrier	Career Entry (Count)	Career Entry (Sources)	Role Transition (Count)	Role Transition (Sources)	Retention (Count)	Retention (Sources)	Driver	Career Entry (Count)	Career Entry (Sources)	Role Transition (Count)	Role Transition (Sources)	Retention (Count)	Retention (Sources)
**Lack of support/ Isolation from team (n=19)**	**10** Mixed-methods studies (n=3), qualitative studies (n=7)	[6,14,30,32,40,43,49] (with transition programmes): [31,38,41]	**8** Qualitative studies (n=6), observational studies (n=2)	[9,19,20,27,28,37,44,45]	**1** Observational study (n=1)	[35]	**Support/ integration from team (n=16)**	**11** Mixed-methods studies (n=3), qualitative studies (n=7), quasi-experimental study (n=1)	[6,8,14,32,49] with transition programmes): [24,25,31,38,41,53]	**5** Qualitative studies (n=4), observational studies (n=1)	[9,20,28,39,44]	0	-
**Fear of making mistakes/ self-doubt (n=15)**	**9** Mixed-methods studies (n=2), qualitative studies (n=7)	[10,14,30,32,40,49] (with transition programmes): [1,13,41]	**6** Qualitative studies (n=6)	[19,20,36,39,44,45]	0	-	**Acceptance of making mistakes (n=1)**	**1** Mixed-methods study (n=1)	[32]	0	-	0	-
**Less reflection (n=1)**	**1** Qualitative studies (n=1)	[8]	0	-	0	-	**Getting feedback /reflection (n=8)**	**7** Qualitative studies (n=3), mixed-methods studies (n=3), quasi-experimental study (n=1)	[6,8,32,49] (with transition programmes): [13,38,53]	**1** Qualitative studies (n=1)	[44]	0	-

Attachment 3 to Müller J, Breitinger F, Bräuer P, Dauer B, Zilenzinski M, Paulicke D, Jahn P. *Drivers and barriers to career entry and retention of nurses with initial higher education: a scoping review*. GMS J Med Educ. 2025;42(3):Doc35. DOI: 10.3205/zma001759

Barrier	Career Entry (Count)	Career Entry (Sources)	Role Transition (Count)	Role Transition (Sources)	Reten-tion (Count)	Retention (Sources)	Driver	Career Entry (Count)	Career Entry (Sources)	Role Transition (Count)	Role Transition (Sources)	Retention (Count)	Retention (Sources)
**Lack of experience with complex cases/ new situations (n=2)**	**2** Mixed-methods studies (n=1), qualitative studies (n=1)	With transition programmes: [31,41]	0	-	0	-	**Increa-sing ex-perience (n=1)**	**1** Qualitative studies (n=1)	[8]	0	-	0	-
**Lack of knowledge /less pre-paration through education (n=2)**	**1** Qualitative studies (n=1)	[43]	0	-	**1** Obser-vational studies (n=1)	[35]	**Desire to train and learn (n=4)**	**2** Mixed-methods studies (n=2)	With transition pro-grammes: [25,38]	**2** Qualitative studies (n=2)	[20,37]	0	-
**Lack of job satifaction (n=2)**	0	-	0	-	**2** Qualita-tive studies (n=1), Obser-vational studies (n=1)	[7,35]	**Job variety and satifac-tion (n=1)**	0	-	0	-	**1** Qualita-tive studies (n=1)	[34]
**High workload/ overload (n=13)**	**12** Mixed-methods studies (n=3), qualitative studies (n=9)	[10,14,30,32,40,43,49] With transition pro-grammes: [1,18,31,38, 41]	0	-	**1** Ober-vational studies (n=1)	[35]	**Integration into work enviro-ments (n=15)**	**11 Mixed-methods studies (n=5), qualitative studies (n=6)**	[6,10,32,40, 49] With transition pro-grammes: [13,18,24,25,31,38]	**4** Qualitative studies (n=4)	[19,37,39,45]		

Attachment 3 to Müller J, Breitinger F, Bräuer P, Dauer B, Zilenzinski M, Paulicke D, Jahn P. *Drivers and barriers to career entry and retention of nurses with initial higher education: a scoping review*. GMS J Med Educ. 2025;42(3):Doc35. DOI: 10.3205/zma001759

Barrier	Career Entry (Count)	Career Entry (Sources)	Role Transition (Count)	Role Transition (Sources)	Reten-tion (Count)	Retention (Sources)	Driver	Career Entry (Count)	Career Entry (Sources)	Role Transition (Count)	Role Transition (Sources)	Retention (Count)	Retention (Sources)
**Tension with medical staff (n=6)**	**4** Mixed-methods studies (n=1), qualitative studies (n=3)	[6,14,32,49]	**2** Qualitative studies (n=1), ob-servational studies (n=1)	[28,44]	0	-	**Support from mentors (n=13)**	**10** Mixed-methods studies (n=4), qualitative studies (n=5), quais-experiment al studies (n=1)	[6,10,14,32, 40,49] With transition pro-grammes:[1 3,25,31,53]	**3** Qualitative studies (n=3)	[29,39,44]	0	-
**Lack of time (n=6)**	**6** Mixed-methods studies (n=1), qualitative studies (n=5)	[6,14,32,40, 43,49]	0	-	0	-							
**Lack of staff (n=5)**	**3** Mixed methods studies (n=1), qualitative studies (n=2)	[6,14,32]	**2** Qualitative studies (n=2)	[19,37]	0	-							
**No say in organi-sation (n=4)**	**4** Qualitative studies (n=4)	[8,9,14,30]	0	-	0	-							

Barrier	Career Entry (Count)	Career Entry (Sources)	Role Transition (Count)	Role Transition (Sources)	Reten-tion (Count)	Retention (Sources)	Driver	Career Entry (Count)	Career Entry (Sources)	Role Transition (Count)	Role Transition (Sources)	Retention (Count)	Retention (Sources)
**Violence in organi-sations (n=2)**	**2** Qualitative studies (n=2)	[14,40]	0	-	0	-							
**Lack of challenge (n=1)**	0	-	0	-	**1** Obser-vational studies (n=1)	[35]							
**Work-life-imbalance (n=1)**	0	-	0	-	**1** Obser-vational studies (n=1)	[35]							
